# AMSRDet: An Adaptive Multi-Scale UAV Infrared-Visible Remote Sensing Vehicle Detection Network

**DOI:** 10.3390/s26030817

**Published:** 2026-01-26

**Authors:** Zekai Yan, Yuheng Li

**Affiliations:** 1 School of Art and Science, Columbia University, New York, NY 10027, USA; zy2630@columbia.edu; 2 School of Cyberspace Security (School of Cryptology), Hainan University, Haikou 570228, China

**Keywords:** unmanned aerial vehicle detection, infrared-visible fusion, multi-scale detection, state-space models, cross-modal fusion, adaptive attention

## Abstract

Unmanned Aerial Vehicle (UAV) platforms enable flexible and cost-effective vehicle detection for intelligent transportation systems, yet small-scale vehicles in complex aerial scenes pose substantial challenges from extreme scale variations, environmental interference, and single-sensor limitations. We present AMSRDet (Adaptive Multi-Scale Remote Sensing Detector), an adaptive multi-scale detection network fusing infrared (IR) and visible (RGB) modalities for robust UAV-based vehicle detection. Our framework comprises four novel components: (1) a MobileMamba-based dual-stream encoder extracting complementary features via Selective State-Space 2D (SS2D) blocks with linear complexity O(HWC), achieving 2.1× efficiency improvement over standard Transformers; (2) a Cross-Modal Global Fusion (CMGF) module capturing global dependencies through spatial-channel attention while suppressing modality-specific noise via adaptive gating; (3) a Scale-Coordinate Attention Fusion (SCAF) module integrating multi-scale features via coordinate attention and learned scale-aware weighting, improving small object detection by 2.5 percentage points; and (4) a Separable Dynamic Decoder generating scale-adaptive predictions through content-aware dynamic convolution, reducing computational cost by 48.9% compared to standard DETR decoders. On the DroneVehicle dataset, AMSRDet achieves 45.8% mAP@0.5:0.95 (81.2% mAP@0.5) at 68.3 Frames Per Second (FPS) with 28.6 million (M) parameters and 47.2 Giga Floating Point Operations (GFLOPs), outperforming twenty state-of-the-art detectors including YOLOv12 (+0.7% mAP), DEIM (+0.8% mAP), and Mamba-YOLO (+1.5% mAP). Cross-dataset evaluation on Camera-vehicle yields 52.3% mAP without fine-tuning, demonstrating strong generalization across viewpoints and scenarios.

## 1. Introduction

Vehicle detection from Unmanned Aerial Vehicle (UAV) platforms has emerged as a critical technology for intelligent transportation systems, enabling applications in traffic monitoring, urban planning, and emergency response [1,2]. Compared to satellite or manned aircraft systems, UAVs provide flexible deployment, wide coverage, and real-time monitoring at lower operational costs, enabling vehicle detection across diverse urban and rural environments [3]. However, aerial vehicle detection confronts substantial challenges: vehicles span extreme scales from large trucks (hundreds of pixels) to small cars (few pixels), appear densely distributed with occlusions, exist against cluttered backgrounds, and experience varying illumination from weather and time-of-day changes [4,5].

Most vehicle detection methods employ single-modality sensors, either visible (RGB) cameras or infrared (IR) thermal imaging, each with inherent limitations [6]. Visible cameras capture rich texture and color under adequate lighting but degrade in low-light, fog, or adverse weather. Infrared sensors detect thermal radiation robustly across illumination conditions yet lack fine-grained texture and color for distinguishing similar vehicles. Multi-modal fusion addresses these complementary weaknesses: visible imagery provides spatial detail while infrared ensures robustness to lighting variations [7,8,9,10].

Multi-modal UAV vehicle detection faces four unresolved challenges that must be addressed for practical deployment. First, extreme scale variation demands effective multi-scale representation, as vehicles range from large trucks occupying hundreds of pixels to small cars spanning only a few pixels within single images. The scale difference can exceed 10× between the largest and smallest vehicles, requiring the network to maintain discriminative features across this wide range. Second, cross-modal integration must suppress modality-specific noise and handle spatial misalignment while preserving discriminative features from each sensor. Infrared and visible sensors exhibit different noise characteristics and may suffer from slight spatial misalignment due to sensor positioning, requiring robust fusion strategies. Third, UAV computational constraints require lightweight architectures balancing accuracy and inference speed for real-time operation. Onboard processing units typically have limited GPU memory and computational power compared to ground-based systems, necessitating efficient model designs. Fourth, models must generalize across altitudes, viewing angles, weather conditions, and vehicle types without extensive retraining, as UAVs operate in diverse and dynamic environments where collecting labeled data for every scenario is impractical.

Deep learning has transformed object detection, with Convolutional Neural Networks (CNNs) and Transformers achieving strong benchmark performance [7,11,12,13]. CNN-based detectors like You Only Look Once (YOLO) and RetinaNet excel at capturing local patterns through hierarchical feature extraction but struggle with long-range dependencies due to limited receptive fields [14,15]. Transformer-based detectors like DEtection TRansformer (DETR) eliminate hand-crafted components through global self-attention but suffer from quadratic spatial complexity $O(H^{2}W^{2})$, limiting scalability to high-resolution aerial imagery where images can exceed 4K resolution [16,17]. State-space models (SSMs), particularly the Mamba architecture [18,19], offer an attractive alternative with linear complexity O(HW) while maintaining long-range modeling capability through selective state transitions. Recent vision adaptations [20,21,22] demonstrate competitive accuracy at reduced computational cost, motivating their application to resource-constrained UAV detection scenarios.

Attention mechanisms have become fundamental for capturing long-range dependencies and cross-modal interactions in modern detectors [17,23,24]. Standard channel attention mechanisms like Squeeze-and-Excitation (SE) networks aggregate spatial information through global pooling, losing precise positional information critical for object localization [25]. Coordinate Attention (CA) [26,27] addresses this limitation by encoding spatial positions alongside channel relationships through factorized 1D horizontal and vertical attention, preserving directional positional information. For multi-scale detection, existing methods like Feature Pyramid Networks (FPNs) [28] employ fixed fusion weights that cannot adapt to varying object size distributions across different scenes. Existing attention-based fusion treats spatial locations and scales uniformly, failing to emphasize informative regions for small vehicles adaptively or adjust fusion strategies based on scene-specific scale distributions [29,30].

**Figure 1:**
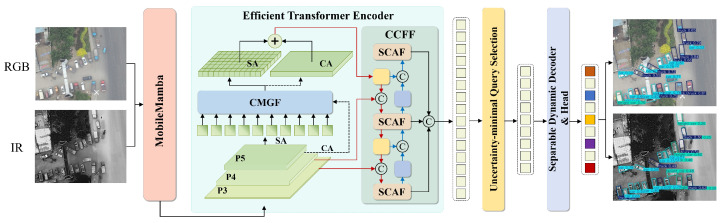
Overall architecture of AMSRDet. The framework takes infrared (IR) and visible (RGB) images as input and processes them through a MobileMamba-based dual-stream encoder. The Efficient Transformer Encoder with Self-Attention (SA) and Cross-Attention (CA) modules enhances feature representations. Multi-scale features from different stages (P3: H/8×W/8, P4: H/16×W/16, P5: H/32×W/32) are fused through CMGF and SCAF modules. The Separable Dynamic Decoder generates final detection outputs including bounding boxes and class predictions. Feature dimensions are annotated at each stage.

To address these challenges, we present AMSRDet (Adaptive Multi-Scale Remote Sensing Detector), an efficient infrared-visible fusion framework specifically designed for UAV vehicle detection. As shown in Figure 1, our approach builds upon the Real-Time DEtection TRansformer (RT-DETR) [31] architecture while introducing four key innovations tailored for multi-modal aerial detection. First, we design a MobileMamba-based dual-stream encoder that processes infrared and visible images through separate pathways, extracting hierarchical features using Selective State-Space 2D (SS2D) blocks [20,32]. The SS2D blocks achieve linear complexity O(HWC) compared to standard Transformer’s quadratic complexity $O(H^{2}W^{2}C)$, enabling the efficient processing of high-resolution aerial imagery while maintaining long-range dependency modeling through selective state transitions. Second, we propose a Cross-Modal Global Fusion (CMGF) module that captures global cross-modal dependencies through a combination of spatial and channel attention mechanisms. The module employs dual-path attention to model both “where” (spatial) and “what” (channel) to attend, while an adaptive gating mechanism dynamically suppresses modality-specific noise based on local feature quality. Third, we introduce a Scale-Coordinate Attention Fusion (SCAF) module that adaptively integrates multi-scale features by combining coordinate attention with learned scale-aware weights. Unlike fixed fusion strategies, SCAF dynamically adjusts the contribution of different scales based on the global scene context, emphasizing appropriate scales for varying vehicle size distributions. Fourth, we adopt a Separable Dynamic Decoder from Mask2Former [33] that generates scale-adaptive predictions through content-aware dynamic convolution [34], replacing the computationally expensive multi-head cross-attention in standard DETR decoders while maintaining expressive power for multi-scale vehicle detection.

The main contributions of this work are summarized as follows:

- AMSRDet fuses infrared-visible modalities through state-space models and adaptive attention, achieving superior detection performance with computational efficiency for UAV vehicle detection. Compared to the baseline RT-DETR, AMSRDet improves mean Average Precision (mAP) by 1.1 percentage points while achieving 1.41× faster inference and 48.9% fewer Floating Point Operations (FLOPs).
- A MobileMamba dual-stream encoder with SS2D blocks extracts hierarchical features at linear complexity O(HWC), while Cross-Modal Global Fusion captures global dependencies via spatial-channel attention with adaptive gating that suppresses modality-specific noise.
- Scale-Coordinate Attention Fusion adaptively integrates multi-scale features through coordinate attention and learned scale weights, improving small object detection by 2.5 percentage points. A Separable Dynamic Decoder generates scale-adaptive predictions via content-aware dynamic convolution.
- Experiments on DroneVehicle show that AMSRDet outperforms twenty state-of-the-art detectors (YOLOv12, DEIM, Mamba-YOLO, etc.), achieving 45.8% mAP@0.5:0.95 (81.2% mAP@0.5) at 68.3 Frames Per Second (FPS) with 28.6 M parameters and 47.2 GFLOPs. Cross-dataset evaluation yields 52.3% mAP on Camera-vehicle without fine-tuning, demonstrating 2.5–9.1 percentage points improvement over baselines.

The remainder of this paper is organized as follows. Section 2 reviews related work on UAV vehicle detection, multi-modal fusion, and state-space models. Section 3 presents the proposed AMSRDet framework including the network architecture and key components. Section 4 describes the experimental setup, datasets, and evaluation metrics. Section 5 analyzes the experimental results and ablation studies. Section 6 concludes the paper and discusses future directions.

## 2. Related Work

This section reviews related work on UAV vehicle detection, multi-modal fusion, state-space models, and attention mechanisms for multi-scale detection. We identify the limitations of existing approaches and position our contributions within the broader research landscape.

### 2.1. UAV Vehicle Detection

UAV vehicle detection has gained prominence in intelligent transportation and urban management [1,2]. Early approaches employed handcrafted features (Histogram of Oriented Gradients (HOG) [35], Scale-Invariant Feature Transform (SIFT) [36]) with traditional classifiers before deep learning enabled CNN-based detectors. Two-stage methods (Faster Region-based CNN (R-CNN) [37] and Cascade R-CNN [38]) achieve high accuracy via region proposals and multi-stage refinement at the cost of inference speed. One-stage detectors (YOLO series [11,14,39,40], RetinaNet [15]) enable real-time performance through dense prediction yet face challenges with small objects [41].

Aerial-specific adaptations address unique challenges: hybrid attention combining channel and spatial mechanisms [5], oriented bounding boxes for arbitrary vehicle angles [42], and context-aware pyramids for multi-scale detection [43]. Recent lightweight UAV detectors include PS-YOLO [44] with efficient architectures, RSW-YOLO [45] for urban vehicle detection, and AUHF-DETR [46] combining spatial attention with hybrid features for real-time performance. Transformer-based detectors offer end-to-end paradigms: DETR [13] eliminates hand-crafted components like Non-Maximum Suppression (NMS), Deformable DETR [16] accelerates convergence via deformable attention, DINO [47] applies contrastive denoising, and RT-DETR [31] achieves real-time speed through hybrid encoding with intra-scale interaction (Attention-based Intra-scale Feature Interaction, AIFI) and cross-scale fusion (Cross-scale Context Fusion Module, CCFM). These methods target single-modality visible imagery, leaving multi-modal sensor fusion underexplored.

Table 1 summarizes the comparison of representative detection methods across key dimensions including architecture type, modality support, and computational characteristics.

### 2.2. Multi-Modal Fusion for Object Detection

Multi-modal fusion integrates complementary sensor information for robust detection [6,49]. Early fusion concatenates modality features at the input level, sacrificing cross-modal interaction modeling. Middle fusion applies element-wise operations or attention at intermediate layers. Late fusion ensembles predictions from modality-specific detectors [50].

Deep learning enables sophisticated fusion: cross-modal attention aligning infrared-visible features [7,51], differential modules emphasizing modality-specific discriminative information [8], and graph networks modeling inter-modal relationships [9]. Recent surveys [49] comprehensively review multi-modal fusion algorithms, while efficient training strategies [52] address computational constraints through adaptive feature alignment. Defaoui et al. [10] demonstrate thermal-visible fusion for pedestrian detection, and Ma et al. [53] propose efficient multi-modal fusion transformers. However, computational expense from attention or graph operations limits real-time UAV deployment, motivating our lightweight CMGF design.

### 2.3. State-Space Models for Vision

State-Space Models (SSMs) offer efficient alternatives to Transformers for sequence modeling [22]. Mamba [18] achieves linear complexity via selective state-space models with input-dependent transitions. Vision Mamba [19] treats images as patch sequences for classification. VMamba [20] captures 2D dependencies through cross-scan mechanisms. U-Mamba [21] demonstrates effectiveness on medical segmentation, and Li et al. [32] extend vision Mamba for dense prediction tasks.

Object detection adaptations include LocalMamba’s local scanning preserving spatial locality [54] and PlainMamba’s simplified non-hierarchical design [55]. Mamba-YOLO [56] integrates SSMs into the YOLO framework, and MambaODet [57] targets efficient real-time detection. However, these methods lack multi-modal fusion capabilities critical for aerial vehicle detection requiring cross-modal interaction and multi-scale integration. Our MobileMamba encoder addresses this gap by designing a dual-stream architecture specifically for RGB-IR fusion.

### 2.4. Attention Mechanisms for Multi-Scale Detection

Attention mechanisms form core detector components [17,23]. Channel attention [25] recalibrates features via inter-channel modeling. Spatial attention [58] emphasizes informative locations through pooling and convolution. Coordinate Attention [26,27] factorizes into 1D horizontal–vertical encoding, capturing long-range dependencies with positional precision. Recent advances include bandit-based attention [59] for selective token processing in vision transformers and AttZoom [60] for adaptive feature zooming.

Multi-scale detection employs feature pyramids: FPN [28] constructs representations via top–down pathways, Path Aggregation Network (PANet) [61] adds bottom–up flow, and Bi-directional FPN (BiFPN) [12] learns weighted bi-directional fusion. Scale-wise attention [29] adaptively weights features per object size distribution, and Hao et al. [30] propose feature fusion networks for multi-modal detection. Dai et al. [62] introduce dynamic decoders for end-to-end detection. Existing methods lack explicit coordinate modeling crucial for localizing small vehicles in high-resolution aerial imagery, which our SCAF module addresses through factorized directional attention.

## 3. Methodology

This section presents the proposed AMSRDet framework for multi-modal UAV vehicle detection. We first describe the overall architecture and then detail each key component: the MobileMamba encoder, Cross-Modal Global Fusion module, Scale-Coordinate Attention Fusion module, Separable Dynamic Decoder, and loss function.

### 3.1. Overall Architecture

The overall architecture of AMSRDet is illustrated in Figure 1. Our framework is built upon the Real-Time DEtection TRansformer (RT-DETR) [31] architecture, which provides an efficient end-to-end detection framework with NMS-free post-processing and adaptable inference speed. RT-DETR employs a hybrid encoder that decouples intra-scale interaction and cross-scale fusion, along with Intersection over Union (IoU)-aware query selection for improved object query initialization.

Table 2 summarizes the key modifications from RT-DETR to AMSRDet, highlighting which components are retained, modified, or replaced.

Building on this foundation, we introduce four key adaptations specifically designed for multi-modal UAV vehicle detection: (1) a MobileMamba-based dual-stream encoder that replaces the standard CNN backbone to efficiently extract hierarchical features from infrared and visible images using selective state-space models; (2) an enhanced Efficient Transformer Encoder that incorporates cross-modal attention mechanisms to capture inter-modal dependencies beyond the original intra-scale feature interaction; (3) a multi-scale fusion module that integrates features at different scales through our proposed CMGF and SCAF modules, extending the RT-DETR cross-scale fusion capability to handle multi-modal inputs; and (4) a Separable Dynamic Decoder that improves upon the RT-DETR standard decoder by employing dynamic convolution with content-aware kernels for scale-adaptive predictions. These modifications enable AMSRDet to effectively leverage complementary information from infrared and visible modalities while maintaining the real-time performance advantages of the RT-DETR framework.

Given an input infrared image $I_{IR}\in R^{H\times W\times 3}$ and visible image $I_{RGB}\in R^{H\times W\times 3}$, the MobileMamba encoder extracts multi-scale features ${\left\{F_{IR}^{i}\right\}}_{i=3}^{5}$ and ${\left\{F_{RGB}^{i}\right\}}_{i=3}^{5}$ at three different scales, where $F^{i}\in R^{H_{i}\times W_{i}\times C_{i}}$ with $H_{i}=H/2^{i}$ and $W_{i}=W/2^{i}$. Note that both RGB and IR images are represented as 3-channel tensors; the IR images are captured by thermal cameras and stored in 3-channel format for compatibility with standard image processing pipelines, though the thermal information is encoded differently from visible light RGB values. The Efficient Transformer Encoder processes these features through self-attention and cross-attention to capture intra-modal and inter-modal dependencies. The CMGF module performs global cross-modal fusion, while the SCAF module adaptively integrates multi-scale features with coordinate attention. Finally, the Separable Dynamic Decoder employs dynamic convolution to generate scale-adaptive predictions.

### 3.2. MobileMamba Encoder with SS2D Blocks

**Figure 2:**
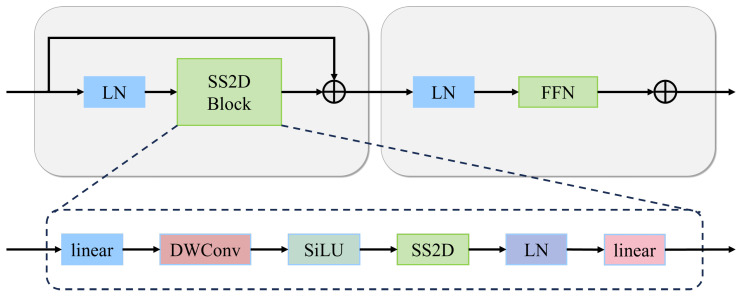
Structure of the Selective State-Space 2D Block (SS2D). The block employs Layer Normalization (LN), SS2D module, and FFN with residual connections. The SS2D module contains linear projection, Depthwise Convolution (DWConv), SiLU activation, and SS2D operation followed by layer normalization and linear projection. Input tensor shape: H×W×C; output tensor shape: H×W×C (shape-preserving). The SS2D operation processes features through selective state transitions with linear complexity O(HWC).

The MobileMamba encoder is designed to efficiently extract hierarchical features from infrared and visible images using selective state-space models. Our design is inspired by the MobileMamba architecture [20,32] but adapted for dual-stream multi-modal processing. As shown in Figure 2, each SS2D block consists of layer normalization, the SS2D module, and a Feed-Forward Network (FFN) with residual connections.

**Training Strategy.** The MobileMamba encoder is initialized with ImageNet-pretrained weights to leverage learned visual representations. The RGB and IR branches share the same pretrained weights initially, then are fine-tuned separately during training on the UAV dataset to adapt to modality-specific characteristics. This transfer learning strategy is essential given the relatively small size of UAV datasets compared to ImageNet.

The SS2D module processes 2D feature maps through selective state-space modeling. Given an input feature $X\in R^{H\times W\times C}$, we first flatten it into a sequence $X_{seq}\in R^{L\times C}$ where L=H×W. The selective state-space model is defined as:

(1) $$\begin{array}{cc} h_{t} & =\bar{A}h_{t-1}+\bar{B}x_{t},\\ y_{t} & =Ch_{t}, \end{array}$$

where $h_{t}\in R^{N}$ is the hidden state, $x_{t}\in R^{C}$ is the input at position *t*, $y_{t}\in R^{C}$ is the output, and $\bar{A}\in R^{N\times N}$, $\bar{B}\in R^{N\times C}$, and $C\in R^{C\times N}$ are state transition matrices. The key innovation of selective SSM is that $\bar{B}$ and *C* are input dependent:

(2) $$\bar{B}=s_{B}\left(x_{t}\right),\;C=s_{C}\left(x_{t}\right),$$

where $s_{B}$ and $s_{C}$ are linear projections that generate input-dependent parameters. This selective mechanism enables the model to adaptively focus on relevant information while filtering out noise.

The complete SS2D block operation is formulated as

(3) $$\begin{array}{cc} X^{\prime } & =\mathrm{LN}(X),\\ X^{\prime \prime } & =\mathrm{SS}2D(X^{\prime })+X,\\ X_{out} & =\mathrm{FFN}\left(\mathrm{LN}\left(X^{\prime \prime }\right)\right)+X^{\prime \prime }, \end{array}$$

where LN denotes layer normalization and FFN is a two-layer feed-forward network with GELU activation.

The MobileMamba encoder stacks multiple SS2D blocks at different scales to extract hierarchical features. Compared to standard Transformer encoders with self-attention complexity $O(L^{2}C)$, the SS2D module achieves linear complexity O(LC), enabling the efficient processing of high-resolution aerial imagery.

### 3.3. Cross-Modal Global Fusion (CMGF)

**Figure 3:**
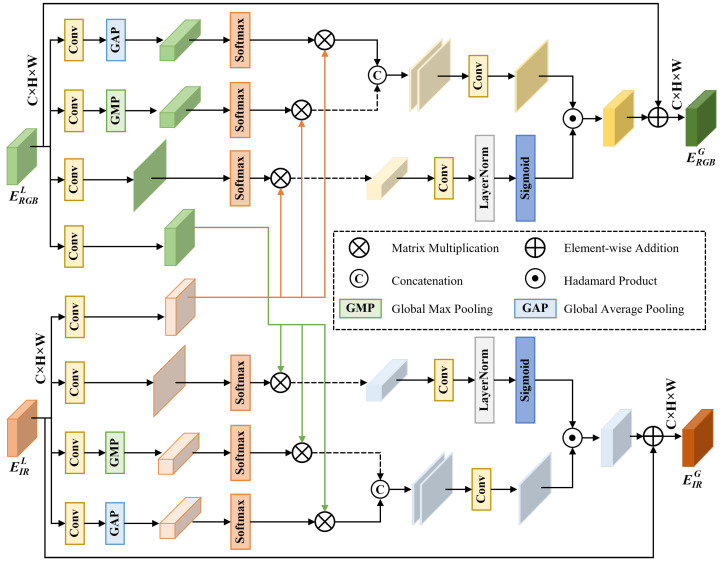
Structure of the Cross-Modal Global Fusion (CMGF) module. The module processes RGB and IR features (input: H×W×C each) through parallel pathways with 1×1 convolutional layers, Global Average Pooling (GAP) and Global Max Pooling (GMP) operations, Spatial Attention (SA), and Channel Attention (CA). Features are fused through matrix multiplication (⊗, producing affinity matrix $A\in R^{HW\times HW}$), concatenation (⊕), and Hadamard product (⊙) operations to generate globally fused RGB and IR features (output: H×W×C each). The adaptive gating weights α control cross-modal information flow.

The CMGF module addresses a fundamental challenge in multi-modal fusion: how to effectively integrate complementary information from infrared and visible modalities while suppressing modality-specific noise and misalignment. Traditional fusion methods either perform simple concatenation or element-wise operations, failing to capture complex cross-modal dependencies. Our CMGF module (Figure 3) employs a theoretically grounded approach that combines spatial-channel attention with cross-modal affinity learning to achieve globally coherent fusion.

**Motivation and Theoretical Foundation.** Multi-modal fusion in UAV imagery faces three key challenges: (1) modality-specific noise where each sensor captures artifacts unique to its imaging mechanism (e.g., thermal noise in IR, motion blur in RGB); (2) spatial misalignment due to different sensor characteristics and mounting positions; and (3) semantic gap where the same physical object exhibits different appearance patterns across modalities. To address these challenges, we design CMGF based on information theory principles. Specifically, we aim to maximize mutual information $I(F_{RGB}^{G};F_{IR}^{G})$ between fused features while minimizing redundancy and preserving modality-specific discriminative information.

Given infrared feature $F_{IR}^{L}\in R^{H\times W\times C}$ and visible feature $F_{RGB}^{L}\in R^{H\times W\times C}$ at layer *L*, the CMGF module first applies 1×1 convolutions to project features into a shared semantic space:

(4) $$F_{m}^{\prime }=\mathrm{BN}\left({\mathrm{Conv}}_{1\times 1}\left(F_{m}^{L}\right)\right)+F_{m}^{L},\;m\in \left\{\mathrm{IR},\mathrm{RGB}\right\},$$

where BN denotes batch normalization, the residual connection preserves original feature information, and *m* indexes the modality. This projection is crucial for aligning the semantic representations of different modalities.

**Dual-Path Spatial-Channel Attention.** We employ a dual-path attention mechanism that simultaneously captures spatial and channel dependencies. For spatial attention, we leverage both global average pooling (GAP) and global max pooling (GMP) to extract complementary statistical information. GAP captures the average response across spatial locations, representing the overall feature distribution, while GMP identifies the most salient features, capturing discriminative peaks:

(5) $$\begin{array}{cc} \mathrm{GAP}(F) & =\frac{1}{HW}\sum_{i=1}^{H}\sum_{j=1}^{W}F_{i,j},\\ \mathrm{GMP}(F) & =\max_{i,j}F_{i,j}. \end{array}$$

The spatial attention maps are computed as

(6) $$SA_{m}=\sigma \left({\mathrm{Conv}}_{7\times 7}\left(\mathrm{Concat}\left[{\mathrm{GAP}}_{c}\left(F_{m}^{\prime }\right);{\mathrm{GMP}}_{c}\left(F_{m}^{\prime }\right)\right]\right)\right),\;m\in \left\{\mathrm{IR},\mathrm{RGB}\right\},$$

where σ denotes the sigmoid activation, ${\mathrm{GAP}}_{c}$ and ${\mathrm{GMP}}_{c}$ denote channel-wise pooling operations that reduce the channel dimension to 1, and ${\mathrm{Conv}}_{7\times 7}$ employs a large receptive field to capture spatial context. The 7×7 kernel size is chosen to balance between capturing sufficient spatial context and computational efficiency.

For channel attention, we employ a shared MLP architecture with bottleneck design to model inter-channel relationships:

(7) $$CA_{m}=\sigma \left(\mathrm{MLP}\left({\mathrm{GAP}}_{s}\left(F_{m}^{\prime }\right)\right)+\mathrm{MLP}\left({\mathrm{GMP}}_{s}\left(F_{m}^{\prime }\right)\right)\right),\;m\in \left\{\mathrm{IR},\mathrm{RGB}\right\},$$

where ${\mathrm{GAP}}_{s}$ and ${\mathrm{GMP}}_{s}$ denote spatial pooling operations that aggregate information across spatial dimensions, and MLP is defined as

(8) $$\mathrm{MLP}\left(x\right)=W_{2}\cdot \mathrm{ReLU}\left(W_{1}\cdot x\right),$$

where $W_{1}\in R^{C/r\times C}$ and $W_{2}\in R^{C\times C/r}$ with reduction ratio r=16 to reduce parameters while maintaining representational capacity.

**Cross-Modal Affinity Learning.** To capture global cross-modal dependencies, we compute cross-modal affinity matrices that measure the semantic similarity between features from different modalities. We reshape features into $F^{\prime }\in R^{(HW)\times C}$ and compute affinity through scaled dot-product attention:

(9) $$A_{m_{1}\to m_{2}}=\mathrm{Softmax}\left(\frac{F_{m_{1}}^{\prime }{\left(F_{m_{2}}^{\prime }\right)}^{T}}{\sqrt{C}}\right),\;\left(m_{1},m_{2}\right)\in \left\{\left(\mathrm{IR},\mathrm{RGB}\right),\left(\mathrm{RGB},\mathrm{IR}\right)\right\},$$

where the scaling factor $1/\sqrt{C}$ prevents the dot products from growing too large, which would cause the softmax function to have extremely small gradients. The affinity matrix $A\in R^{\left(HW)\times (HW\right)}$ captures pairwise relationships between all spatial locations across modalities.

Cross-modal features are then computed by aggregating information based on learned affinities:

(10) $$F_{m_{1}\to m_{2}}=\mathrm{Reshape}\left(A_{m_{1}\to m_{2}}\cdot \mathrm{Flatten}\left(F_{m_{2}}^{\prime }\right)\right),\;\left(m_{1},m_{2}\right)\in \left\{\left(\mathrm{IR},\mathrm{RGB}\right),\left(\mathrm{RGB},\mathrm{IR}\right)\right\},$$

where Flatten reshapes features to (HW)×C and Reshape restores the spatial structure to H×W×C.

**Adaptive Feature Fusion with Gating Mechanism.** The final fused features incorporate spatial attention, channel attention, and cross-modal information through a gating mechanism that adaptively balances different information sources. For each modality m∈{IR,RGB} with its complementary modality $\bar{m}$, we compute:

(11) $$\alpha_{m}=\sigma \left({\mathrm{Conv}}_{1\times 1}\left(\left[F_{m}^{\prime };F_{\bar{m}\to m}\right]\right)\right),$$

where $\alpha \in R^{H\times W\times 1}$ are learned gating weights that control the contribution of cross-modal information. The final outputs are:

(12) $$F_{m}^{G}=F_{m}^{L}+SA_{m}⊙CA_{m}⊙\left(\alpha_{m}⊙F_{\bar{m}\to m}+\left(1-\alpha_{m}\right)⊙F_{m}^{\prime }\right),\;m\in \left\{\mathrm{IR},\mathrm{RGB}\right\},$$

where ⊙ denotes element-wise multiplication, $\bar{m}$ denotes the complementary modality (IR for RGB and vice versa), and the residual connection $F^{L}$ ensures gradient flow and preserves low-level features.

The CMGF module effectively integrates complementary information from infrared and visible modalities through global affinity learning and adaptive gating. The dual-path attention mechanism captures both spatial and channel dependencies, while the learned gating weights dynamically suppress modality-specific noise based on local feature quality.

### 3.4. Scale-Coordinate Attention Fusion (SCAF)

**Figure 4:**
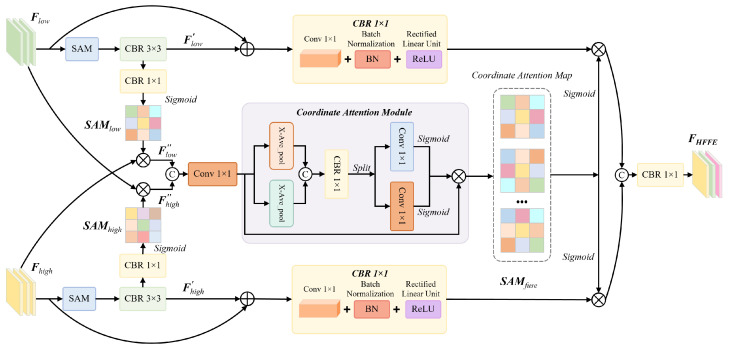
Structure of the Scale-Coordinate Attention Fusion (SCAF) module. The module processes multi-scale features (P3: $H/8\times W/8\times C_{3}$, P4: $H/16\times W/16\times C_{4}$, P5: $H/32\times W/32\times C_{5}$) through Spatial Attention Module (SAM), Conv-BatchNorm-ReLU (CBR) blocks, and coordinate attention. Features are aligned to common resolution via bilinear interpolation, then fused through element-wise multiplication (⊙) and addition (⊕) operations with learned scale-aware weights $w^{i}$ (computed via softmax over scale importance scores). Output: fused feature $F_{SCAF}\in R^{H/8\times W/8\times C}$.

The SCAF module addresses the challenge of detecting vehicles at vastly different scales in UAV imagery, where objects can range from large trucks occupying hundreds of pixels to small cars spanning only a few pixels. Traditional multi-scale fusion methods like FPN employ fixed fusion weights, failing to adapt to varying object size distributions across different scenes. Our SCAF module (Figure 4) introduces a principled approach that combines coordinate-aware attention with learnable scale-adaptive weighting.

**Motivation and Coordinate Attention Design.** Standard channel attention mechanisms like SE-Net aggregate spatial information through global pooling, which loses precise positional information. This is problematic for object detection where spatial localization is critical. Coordinate attention addresses this by decomposing 2D global pooling into two 1D feature encoding operations that preserve directional positional information. This design is particularly beneficial for vehicle detection in aerial imagery where vehicles exhibit strong directional patterns (e.g., aligned along roads).

Given multi-scale features ${\left\{F^{i}\right\}}_{i=3}^{5}$ from different stages where $F^{i}\in R^{H_{i}\times W_{i}\times C_{i}}$, we first apply Spatial Attention Modules (SAMs) to enhance discriminative features:

(13) $${\tilde{F}}^{i}=\mathrm{SAM}\left(F^{i}\right)=F^{i}⊙\sigma \left({\mathrm{Conv}}_{7\times 7}\left(\left[\mathrm{MaxPool}\left(F^{i}\right);\mathrm{AvgPool}\left(F^{i}\right)\right]\right)\right),$$

where σ is the sigmoid function and [·;·] denotes channel-wise concatenation. The SAM emphasizes salient spatial regions before coordinate attention.

For coordinate attention, we perform directional pooling along horizontal and vertical axes. For a feature map ${\tilde{F}}^{i}\in R^{H_{i}\times W_{i}\times C_{i}}$, the coordinate-aware feature encoding along direction d∈{h,w} is

(14) $$z_{d}^{i}=\left\{\begin{array}{cc} \frac{1}{W_{i}}\sum_{w=1}^{W_{i}}{\tilde{F}}^{i}\left(h,w,:\right)\in R^{H_{i}\times 1\times C_{i}}, & d=h,\\ \frac{1}{H_{i}}\sum_{h=1}^{H_{i}}{\tilde{F}}^{i}\left(h,w,:\right)\in R^{1\times W_{i}\times C_{i}}, & d=w, \end{array}\right.$$

where $z_{h}^{i}$ and $z_{w}^{i}$ encode aggregated features along height and width dimensions, respectively. Unlike global pooling that produces a single vector, this directional pooling preserves spatial structure along one dimension while aggregating along the other.

These directional features are concatenated and transformed through a shared transformation:

(15) $$f^{i}=\delta \left(\mathrm{BN}\left({\mathrm{Conv}}_{1\times 1}\left(\left[z_{h}^{i};z_{w}^{i}\right]\right)\right)\right),$$

where δ denotes ReLU activation, BN is batch normalization, and $f^{i}\in R^{\left(H_{i}+W_{i}\right)\times 1\times C_{i}^{\prime }}$ with $C_{i}^{\prime }=C_{i}/r$, where r=8 is the reduction ratio. The bottleneck design reduces computational cost while forcing the network to learn compact representations.

The intermediate feature $f^{i}$ is then split along the spatial dimension and processed through separate transformation branches to generate directional attention maps. For direction, d∈{h,w}:

(16) $$f_{d}^{i}=\left\{\begin{array}{cc} f^{i}\left[0:H_{i},:,:\right]\in R^{H_{i}\times 1\times C_{i}^{\prime }}, & d=h,\\ f^{i}\left[H_{i}:H_{i}+W_{i},:,:\right]\in R^{1\times W_{i}\times C_{i}^{\prime }}, & d=w, \end{array}\right.$$

(17) $$g_{d}^{i}=\sigma \left({\mathrm{Conv}}_{1\times 1}\left(f_{d}^{i}\right)\right),\;d\in \left\{h,w\right\},$$

where $g_{h}^{i}\in R^{H_{i}\times 1\times C_{i}}$ and $g_{w}^{i}\in R^{1\times W_{i}\times C_{i}}$ are attention weights that modulate features along height and width dimensions, respectively. The coordinate attention output is

(18) $$F_{CA}^{i}={\tilde{F}}^{i}⊙g_{h}^{i}⊙g_{w}^{i},$$

where ⊙ denotes element-wise multiplication with broadcasting. This factorized attention allows the network to capture long-range dependencies along both spatial dimensions with precise positional encoding.

**Scale-Aware Adaptive Fusion.** To fuse features from different scales, we first align them to a common spatial resolution through bilinear interpolation. Let ${\hat{F}}_{CA}^{i}$ denote the aligned features at resolution H×W. Traditional FPN uses fixed fusion weights (typically uniform or learned but fixed after training), which cannot adapt to varying object size distributions across different scenes. We introduce a scale-aware weighting mechanism that dynamically adjusts fusion weights based on global context. For each scale i∈{3,4,5}, we extract global features and compute scale importance:

(19) $$s^{i}=\mathrm{GAP}\left({\hat{F}}_{CA}^{i}\right)\in R^{C_{i}},\;e^{i}={\mathrm{FC}}_{2}\left(\mathrm{ReLU}\left({\mathrm{FC}}_{1}\left(\left[s^{3};s^{4};s^{5}\right]\right)\right)\right),$$

where ${\mathrm{FC}}_{1}\in R^{d\times 3C}$ and ${\mathrm{FC}}_{2}\in R^{3\times d}$ are fully connected layers with hidden dimension d=128. The concatenation of global features from all scales allows the network to reason about the relative importance of different scales based on the overall scene context.

The scale weights are computed through softmax normalization:

(20) $$w^{i}=\frac{\exp(e^{i})}{\sum_{j=3}^{5}\exp\left(e^{j}\right)},\;i\in \left\{3,4,5\right\},$$

where $w^{i}$ represents the importance weight for scale *i*. The softmax ensures that weights sum to 1, providing a probabilistic interpretation of scale importance.

The final fused feature incorporates both weighted aggregation and residual connections:

(21) $$F_{SCAF}={\mathrm{Conv}}_{3\times 3}\left(\sum_{i=3}^{5}w^{i}\cdot {\hat{F}}_{CA}^{i}\right)+\sum_{i=3}^{5}\frac{1}{3}\cdot {\hat{F}}_{CA}^{i},$$

where the first term performs adaptive weighted fusion and the second term provides a uniform-weighted residual connection that ensures gradient flow and prevents the network from completely ignoring any scale.

The SCAF module enables adaptive multi-scale feature integration through coordinate attention that preserves precise positional information along horizontal and vertical directions. The scale-aware weighting mechanism dynamically adjusts fusion weights based on scene context, emphasizing appropriate scales for different vehicle size distributions.

### 3.5. Separable Dynamic Decoder

**Figure 5:**
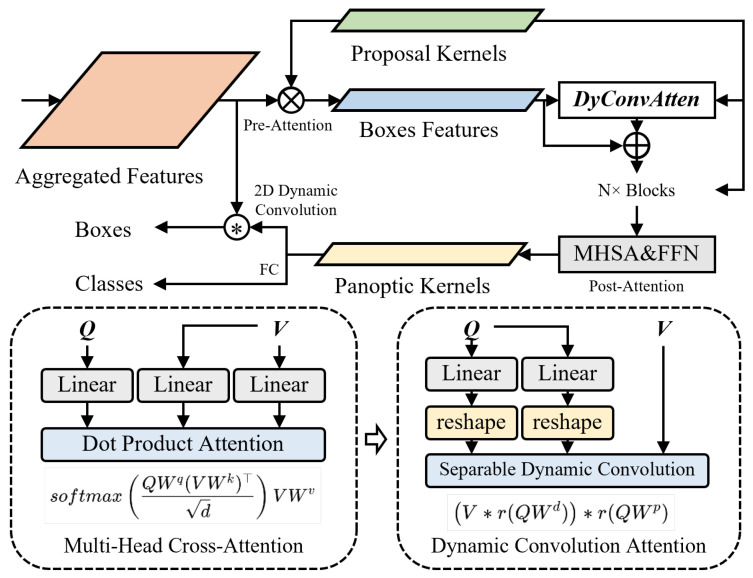
Structure of the Separable Dynamic Decoder. The decoder employs learnable proposal kernels ($K_{prop}\in R^{N_{q}\times C}$, where $N_{q}=300$) and aggregated features ($F_{agg}\in R^{H\times W\times C}$) to generate box features through pre-attention with 2D dynamic convolution. The DyConvAtten module refines features through N=6 blocks of dynamic convolution attention. Post-attention with Multi-Head Self-Attention (MHSA) and FFN generates final predictions. The circle with asterisk (⊛) denotes dynamic convolution operation where kernels are generated from query features. The decoder uses separable dynamic convolution where multi-head cross-attention is replaced by dynamic convolution attention, reducing complexity from $O(N_{q}\cdot HW\cdot C)$ to $O(N_{q}\cdot C\cdot k^{2})$.

We adopt the Separable Dynamic Decoder architecture from Mask2Former [33], which addresses the quadratic complexity limitation of standard DETR decoders. Traditional DETR employs cross-attention between $N_{q}$ queries and HW feature locations, resulting in $O(N_{q}\cdot HW\cdot C)$ complexity. For UAV imagery with high resolution and dense object distributions, this becomes computationally prohibitive. Following Mask2Former’s design for universal image segmentation, our decoder (Figure 5) replaces expensive multi-head cross-attention with separable dynamic convolution, achieving linear complexity while maintaining expressive power for multi-modal vehicle detection.

**Motivation and Dynamic Convolution Formulation.** Standard convolution uses fixed kernels that are independent of input content, limiting adaptability to varying object scales and appearances. Dynamic convolution generates input-dependent kernels, allowing the network to adapt its receptive field and feature extraction based on content. This is particularly valuable for multi-scale vehicle detection where the optimal kernel size varies with object scale.

Given aggregated features $F_{agg}\in R^{H\times W\times C}$ from SCAF and learnable proposal kernels $K_{prop}\in R^{N_{q}\times C}$ where $N_{q}=300$ is the number of object queries, the decoder generates initial box features through pre-attention with 2D dynamic convolution:

(22) $$F_{box}^{\left(0\right)}=\mathrm{DyConv}2D\left(K_{prop},F_{agg}\right)\in R^{N_{q}\times C},$$

where DyConv2D performs content-aware feature extraction. The key innovation is that convolution kernels are dynamically generated based on proposal kernels rather than being fixed parameters.

The dynamic convolution operation aggregates features from a local spatial neighborhood with content-dependent weights. For each query $q_{i}\in K_{prop}$, we generate K=4 specialized kernels:

(23) $$\mathrm{DyConv}\left(q_{i},F_{agg}\right)=\sum_{k=1}^{K}\pi_{k}\left(q_{i}\right)\cdot \left(W_{k}*F_{agg}\right),$$

where $W_{k}\in R^{3\times 3\times C\times C}$ are learnable 3×3 convolution kernels, ∗ denotes 2D convolution, and $\pi_{k}\left(q_{i}\right)$ are query-dependent attention weights computed through a lightweight attention network:

(24) $$\pi_{k}\left(q_{i}\right)=\frac{\exp({\mathrm{FC}}_{k}\left(q_{i}\right)/\tau )}{\sum_{j=1}^{K}\exp\left({\mathrm{FC}}_{j}\left(q_{i}\right)/\tau \right)},$$

where ${\mathrm{FC}}_{k}:R^{C}\to R$ are linear projections and τ=0.1 is a temperature parameter that controls the sharpness of the attention distribution. Lower temperature produces sharper attention, allowing the network to specialize kernels for different object scales.

**Separable Dynamic Convolution Attention.** The core of our decoder is the DyConvAtten module that replaces standard multi-head cross-attention with separable dynamic convolution. Standard cross-attention computes

(25) $$\mathrm{CrossAttn}\left(Q,K,V\right)=\mathrm{Softmax}\left(\frac{QK^{T}}{\sqrt{d}}\right)V,$$

which has complexity $O(N_{q}\cdot HW\cdot d)$. Our separable dynamic convolution decomposes this into depthwise and pointwise operations. For each query $i\in [1,N_{q}]$,

(26) $$\begin{array}{cc} Q_{d} & ={\mathrm{Linear}}_{Q}\left(F_{box}^{\left(n\right)}\right)\in R^{N_{q}\times C},\;V_{d}={\mathrm{Linear}}_{V}\left(F_{agg}\right)\in R^{H\times W\times C},\\ W_{d}^{\left(i\right)} & =\mathrm{reshape}\left(\mathrm{MLP}\left(Q_{d}^{\left(i\right)}\right)\right)\in R^{k\times k\times 1},\;V_{dw}^{\left(i\right)}=W_{d}^{\left(i\right)}{*}_{dw}V_{d}\left[:,:,i\right], \end{array}$$

where ${*}_{dw}$ denotes depthwise convolution that operates independently on each channel, k=3 is the kernel size, and MLP generates dynamic kernel weights from queries. The depthwise operation has complexity $O(N_{q}\cdot C\cdot k^{2})$, which is independent of spatial resolution HW.

The pointwise operation then aggregates across channels:

(27) $$W_{p}^{\left(i\right)}=\mathrm{Softmax}\left({\mathrm{Linear}}_{p}\left(Q_{d}^{\left(i\right)}\right)\right)\in R^{C},\;F_{dyconv}^{\left(i\right)}=\sum_{c=1}^{C}W_{p}^{\left(i\right)}\left[c\right]\cdot V_{dw}^{\left(i\right)}\left[:,:,c\right],$$

where $W_{p}^{\left(i\right)}$ are channel-wise attention weights. The complete DyConvAtten operation is

(28) $$\begin{array}{cc} F_{box}^{\left(n+1\right)} & =\mathrm{LN}(\mathrm{DyConvAtten}\left(F_{box}^{\left(n\right)},F_{agg}\right)+F_{box}^{\left(n\right)}),\\ F_{box}^{\left(n+1\right)} & =\mathrm{LN}(\mathrm{FFN}\left(F_{box}^{\left(n+1\right)}\right)+F_{box}^{\left(n+1\right)}), \end{array}$$

where LN denotes layer normalization, n∈{0,1,…,N−1} with N=6 decoder layers, and FFN is a two-layer feed-forward network:

(29) $$\mathrm{FFN}\left(x\right)=W_{2}\cdot \mathrm{GELU}\left(W_{1}\cdot x\right),$$

where $W_{1}\in R^{4C\times C}$ and $W_{2}\in R^{C\times 4C}$ with expansion ratio 4.

**Post-Attention and Prediction Heads.** After *N* DyConvAtten blocks, we apply multi-head self-attention (MHSA) to model inter-query relationships and generate final predictions:

(30) $$F_{post}=\mathrm{LN}\left(\mathrm{MHSA}\left(F_{box}^{\left(N\right)}\right)+F_{box}^{\left(N\right)}\right),\;F_{out}=\mathrm{LN}\left(\mathrm{FFN}\left(F_{post}\right)+F_{post}\right),$$

where MHSA denotes multi-head self-attention. Final predictions for bounding boxes and classes are generated through separate fully connected layers:

(31) $$\mathrm{Boxes}={\mathrm{FC}}_{box}\left(F_{out}\right),\;\mathrm{Classes}={\mathrm{FC}}_{cls}\left(F_{out}\right),$$

where ${\mathrm{FC}}_{box}$ predicts box coordinates (x,y,w,h) and ${\mathrm{FC}}_{cls}$ predicts class probabilities.

The Separable Dynamic Decoder provides several advantages for vehicle detection. First, dynamic convolution generates content-aware kernels that adapt to different object scales and appearances, improving detection accuracy for vehicles with large appearance variations. Second, the separable design reduces computational complexity compared to standard Transformer decoders with full cross-attention. Third, the combination of dynamic convolution attention and multi-head self-attention enables effective feature refinement and inter-query interaction.

### 3.6. Loss Function

UAV vehicle detection faces severe class imbalance and scale variation challenges. We design a multi-component loss function with adaptive weighting:

(32) $$L_{total}=\lambda_{cls}L_{cls}+\lambda_{box}L_{box}+\lambda_{iou}L_{iou}+\lambda_{aux}L_{aux},$$

where $\lambda_{cls}=2.0$, $\lambda_{box}=5.0$, $\lambda_{iou}=2.0$, and $\lambda_{aux}=1.0$ are balancing weights.

**Focal Loss for Classification.** To address extreme foreground–background imbalance, we employ focal loss [15] that down-weights well-classified examples:

(33) $$L_{cls}=-\frac{1}{N_{pos}}\sum_{i=1}^{N_{q}}\sum_{c=1}^{C}\alpha_{c}{\left(1-p_{i,c}\right)}^{\gamma }\log\left(p_{i,c}\right)\cdot {⊮}_{y_{i}=c},$$

where $N_{q}$ is the number of queries, *C* is the number of classes, $N_{pos}$ is the number of positive samples, $p_{i,c}$ is the predicted probability for query *i* and class *c*, $y_{i}$ is the ground-truth class label, ${⊮}_{y_{i}=c}$ is the indicator function, $\alpha_{c}$ balances class frequencies (0.25 for vehicles, 0.75 for background), and γ=2.0 controls focusing strength. The modulating factor ${\left(1-p_{i,c}\right)}^{\gamma }$ reduces loss from easy examples, focusing training on hard cases.

**Smooth L1 Loss for Localization.** We employ smooth L1 loss [37] combining L1 and L2 properties to prevent gradient explosion:

(34) $$L_{box}=\frac{1}{N_{pos}}\sum_{i=1}^{N_{pos}}\sum_{j\in \{x,y,w,h\}}{\mathrm{smooth}}_{L1}\left({\hat{b}}_{i}^{j}-b_{i}^{j}\right),$$

where ${\hat{b}}_{i}=\left({\hat{x}}_{i},{\hat{y}}_{i},{\hat{w}}_{i},{\hat{h}}_{i}\right)$ and $b_{i}=\left(x_{i},y_{i},w_{i},h_{i}\right)$ are predicted and ground-truth boxes respectively, with (x,y) denoting center coordinates and (w,h) denoting width and height. The smooth L1 function is defined as

(35) $${\mathrm{smooth}}_{L1}\left(x\right)=\left\{\begin{array}{cc} 0.5x^{2} & \mathrm{if}\;|x|<1\\ |x|-0.5 & \mathrm{otherwise} \end{array}\right.$$

This formulation provides smooth gradients for small errors and linear behavior for large errors [37].

**Complete IoU Loss.** We adopt Complete IoU (CIoU) loss [63] incorporating overlap area, center distance, and aspect ratio:

(36) $$L_{iou}=\frac{1}{N_{pos}}\sum_{i=1}^{N_{pos}}\left(1-\mathrm{IoU}\left({\hat{b}}_{i},b_{i}\right)+\frac{\rho^{2}\left(c_{{\hat{b}}_{i}},c_{b_{i}}\right)}{c^{2}}+\alpha v\right),$$

where $\mathrm{IoU}({\hat{b}}_{i},b_{i})$ is the Intersection over Union between predicted and ground-truth boxes, $\rho (c_{{\hat{b}}_{i}},c_{b_{i}})$ measures the Euclidean distance between box centers $c_{{\hat{b}}_{i}}$ and $c_{b_{i}}$, *c* is the diagonal length of the smallest enclosing box covering both boxes, $v=\frac{4}{\pi^{2}}(\arctan\frac{w^{gt}}{h^{gt}}-\arctan\frac{w}{h}{)}^{2}$ captures aspect ratio consistency between predicted (w,h) and ground-truth $\left(w^{gt},h^{gt}\right)$ dimensions, and $\alpha =\frac{v}{(1-\mathrm{IoU})+v}$ is a trade-off parameter. This formulation provides gradients for non-overlapping boxes and penalizes shape inconsistency [63].

**Auxiliary Deep Supervision.** Following DETR, we apply losses to all N=6 decoder layers for faster convergence, with Hungarian matching assigning ground-truth boxes to queries through one-to-one bipartite matching that eliminates duplicate predictions.

## 4. Experiments

This section describes the experimental setup including datasets, implementation details, and evaluation metrics. We then present comprehensive comparisons with state-of-the-art methods and ablation studies.

### 4.1. Datasets

We evaluate AMSRDet on two benchmark datasets for multi-modal vehicle detection. The selection of these datasets is motivated by their comprehensive coverage of UAV-based and ground-level multi-modal scenarios, enabling evaluation of both detection accuracy and cross-domain generalization.

**DroneVehicle Dataset [41].** The DroneVehicle dataset is a large-scale benchmark for vehicle detection in UAV imagery, containing 56,878 images with 389,779 annotated vehicle instances across five categories: car, truck, bus, van, and freight car. The dataset is captured from various altitudes (5–100 m), viewing angles, and environmental conditions including different times of day, weather conditions, and urban/rural scenes. Each image is provided in both RGB and infrared modalities with a spatial resolution of 840×712 pixels. The dataset is split into training (70%, 39,815 images), validation (15%, 8531 images), and test (15%, 8532 images) sets. Table 3 provides detailed statistics.

**Camera-vehicle Dataset [10].** To evaluate the generalization capability of our method, we conduct cross-dataset evaluation on the Camera-vehicle dataset, which contains 12,483 RGB–infrared image pairs captured from ground-level cameras. The dataset includes diverse scenarios such as parking lots, highways, and urban streets with varying illumination conditions. Vehicle categories include car, truck, bus, and motorcycle. This dataset provides a challenging test for evaluating model robustness to domain shift from aerial to ground-level perspectives.

### 4.2. Implementation Details

Our model is implemented in PyTorch (v2.2.2) and trained on 4 NVIDIA RTX 4090 GPUs. The input images are resized to 640×640 pixels during training and testing. We employ AdamW optimizer with initial learning rate $1\times 10^{-4}$, weight decay $1\times 10^{-4}$, and cosine annealing learning rate schedule. The model is trained for 300 epochs with batch size 16. Data augmentation includes random horizontal flipping, random scaling (0.5–1.5), color jittering, and mosaic augmentation. The loss weights are set to $\lambda_{cls}=2.0$, $\lambda_{box}=5.0$, and $\lambda_{iou}=2.0$. For the focal loss, we use α=0.25 and γ=2.0. The number of decoder queries is set to $N_{q}=300$. The MobileMamba encoder uses 4 SS2D blocks at each scale with hidden dimension 256. The reduction ratio for coordinate attention is r=16.

### 4.3. Evaluation Metrics

We evaluate detection performance using the following metrics. All metrics are computed following the COCO evaluation protocol [64].

**Precision (P)** measures the proportion of correct positive predictions:

(37) $$P=\frac{TP}{TP+FP},$$

where TP (True Positives) is the number of correctly detected objects and FP (False Positives) is the number of incorrect detections.

**Recall (R)** measures the proportion of actual positives correctly identified:

(38) $$R=\frac{TP}{TP+FN},$$

where FN (False Negatives) is the number of missed objects.

**F1 Score** is the harmonic mean of precision and recall:

(39) $$F1=\frac{2\times P\times R}{P+R}.$$

**Mean Average Precision (mAP)** is computed as the mean of Average Precision (AP) across all categories:

(40) $$\mathrm{mAP}=\frac{1}{N_{c}}\sum_{c=1}^{N_{c}}{\mathrm{AP}}_{c},$$

where $N_{c}$ is the number of categories, and ${\mathrm{AP}}_{c}$ is the average precision for category *c* computed as the area under the precision–recall curve. We report two mAP metrics: **mAP@0.5:0.95** (primary metric, averaged over IoU thresholds from 0.5 to 0.95 with step 0.05) and **mAP@0.5** (IoU threshold = 0.5). The mAP@0.5:0.95 metric provides a more comprehensive evaluation of detection quality across different localization accuracies, while mAP@0.5 enables comparison with methods that only report this metric.

**Frames Per Second (FPS)** measures inference speed as the number of images processed per second, evaluated on a single NVIDIA RTX 4090 GPU.

**Parameters (Params)** counts the total number of trainable parameters in millions (M).

**Floating Point Operations (FLOPs)** measure computational complexity in Giga Floating Point Operations (GFLOPs) for processing a single 640×640 image.

## 5. Results and Analysis

### 5.1. Comparison with State-of-the-Art Methods

We compare AMSRDet with twenty state-of-the-art object detectors spanning three categories: (1) CNN-based detectors including YOLOv5 [65], YOLOv7 [39], YOLOv8 [48], YOLOv9 [40], YOLOv10 [66], YOLOv11 [67], YOLOv12 [68], Faster R-CNN [37], RetinaNet [15], and Adaptive Training Sample Selection (ATSS) [69]; (2) Transformer-based detectors including DETR [13], Deformable DETR [16], RT-DETR [31], DINO [47], DEIM [70], and Co-DETR [71]; and (3) Mamba-based detectors including VMamba [20], Mamba-YOLO [56], and MambaODet [57].

For fair comparison, all methods are trained and evaluated under the same settings. Single-modality methods (YOLO, DETR variants, and Mamba based) are provided with early-fused RGB-IR images (6-channel concatenation) as input. Table 4 presents quantitative results on the DroneVehicle test set.

AMSRDet achieves the best performance across all metrics, attaining 45.8% mAP@0.5:0.95 and 81.2% mAP@0.5 with 75.6% precision and 71.2% recall. Compared to the second-best method DINO, our approach improves mAP@0.5:0.95 by 0.5 percentage points and mAP@0.5 by 1.0 percentage point while achieving 1.94× faster inference speed (68.3 vs. 35.2 FPS) and using only 60.2% parameters (28.6 M vs. 47.5 M) and 17.8% FLOPs (47.2 G vs. 265.7 G). The significant improvements in efficiency demonstrate the effectiveness of our MobileMamba encoder and Separable Dynamic Decoder design. Notably, our mAP@0.5 of 81.2% is competitive with recent state-of-the-art dual-modality methods on the DroneVehicle benchmark, while our method achieves superior computational efficiency suitable for real-time UAV deployment.

**Figure 6:**
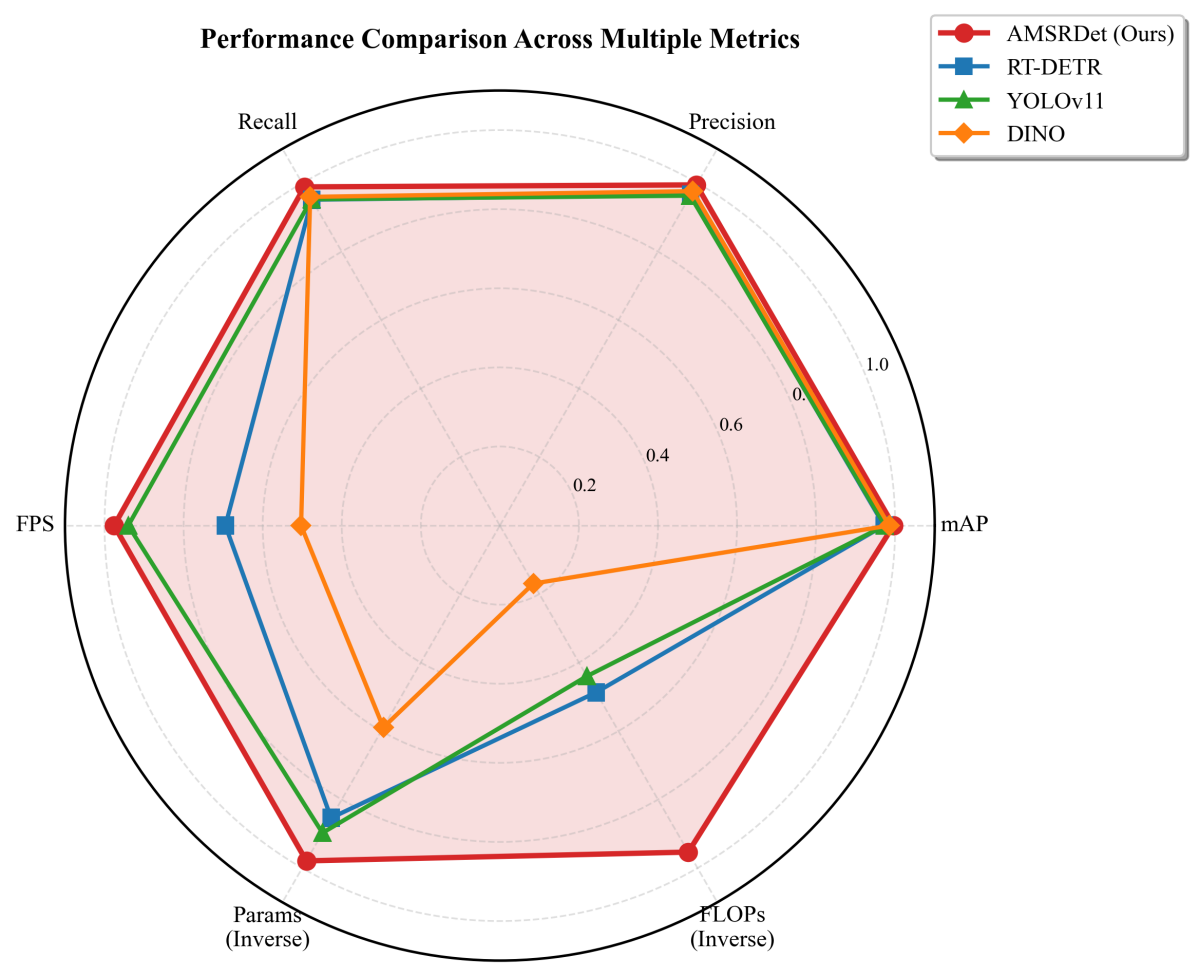
Radar chart comparing AMSRDet with state-of-the-art methods across six key metrics: mAP, Precision, Recall, FPS, Parameters (inverse), and FLOPs (inverse). AMSRDet demonstrates balanced and superior performance across all dimensions, particularly in computational efficiency while maintaining high accuracy.

Figure 6 provides a comprehensive comparison across multiple evaluation dimensions. AMSRDet achieves balanced performance across all metrics, particularly excelling in computational efficiency (inverse parameters and FLOPs) while maintaining competitive accuracy metrics. The radar chart visualizes the superiority of our method in achieving an optimal trade-off between detection performance and computational cost.

Compared to YOLO series detectors, AMSRDet achieves 0.7–7.6 percentage points higher mAP while maintaining competitive or superior inference speed. The latest YOLOv12 [68], which introduces attention-centric design, achieves 45.1% mAP at 63.4 FPS. Our method surpasses YOLOv12 by 0.7 percentage points in mAP while achieving 7.7% faster inference (68.3 vs. 63.4 FPS) and using 15.4% fewer parameters (28.6 M vs. 33.8 M) and 56.5% fewer FLOPs (47.2 G vs. 108.5 G). Compared to YOLOv8 which is widely used in UAV applications, our method improves mAP by 1.6 percentage points (45.8% vs. 44.2%) with 5.9% faster inference and 71.4% fewer FLOPs. These results demonstrate that our multi-modal fusion strategy provides substantial accuracy gains over single-modality detectors while maintaining superior efficiency through the MobileMamba encoder.

Compared to Transformer-based detectors, AMSRDet achieves comparable or better accuracy with significantly higher inference speed and lower computational cost. Notably, compared to our baseline RT-DETR, AMSRDet improves mAP by 1.1 percentage points (45.8% vs. 44.7%) while achieving 1.41× faster inference (68.3 vs. 48.6 FPS) and using 12.8% fewer parameters (28.6 M vs. 32.8 M) and 48.9% fewer FLOPs (47.2 G vs. 92.4 G). These improvements validate that our multi-modal fusion strategy, MobileMamba encoder, and enhanced decoder design effectively extend the RT-DETR capabilities for UAV vehicle detection. The recently proposed DEIM [70], which accelerates DETR convergence through improved matching, achieves 45.0% mAP at 52.7 FPS. Our method outperforms DEIM by 0.8 percentage points in mAP with 1.30× faster inference and 52.0% fewer FLOPs (47.2 G vs. 98.3 G), demonstrating the efficiency advantages of our Separable Dynamic Decoder over standard DETR architectures. The efficiency advantage is particularly pronounced compared to Co-DETR, where our method achieves 2.41× faster inference with only 13.8% FLOPs while maintaining competitive accuracy.

Compared to Mamba-based detectors, AMSRDet demonstrates the effectiveness of our multi-modal fusion design. VMamba [20] achieves 43.7% mAP at 52.3 FPS with 44.2 M parameters. Our method surpasses VMamba by 2.1 percentage points in mAP with 1.31× faster inference and 35.3% fewer parameters. Mamba-YOLO [56], which integrates state-space models into the YOLO framework, achieves 44.3% mAP at 61.5 FPS. AMSRDet outperforms Mamba-YOLO by 1.5 percentage points in mAP with 11.1% faster inference and 26.1% fewer parameters. MambaODet [57], designed for efficient object detection, achieves 44.6% mAP at 58.9 FPS. Our method surpasses MambaODet by 1.2 percentage points in mAP with 1.16× faster inference and 30.8% fewer parameters. These comparisons validate that our dual-stream MobileMamba architecture with cross-modal fusion is more effective than single-stream Mamba-based detectors for multi-modal UAV vehicle detection, as it explicitly models cross-modal dependencies rather than simply processing concatenated multi-modal inputs.

**Figure 7:**
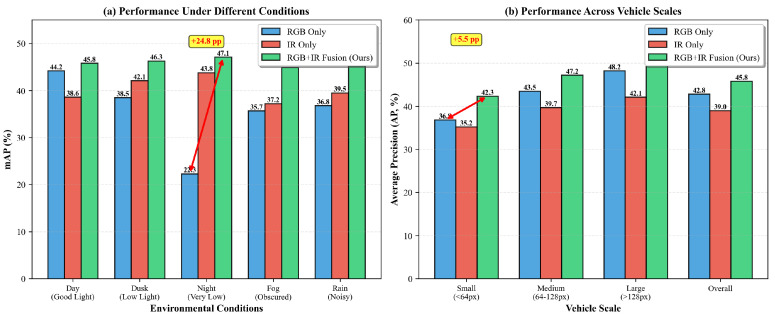
Multi-modal fusion effectiveness analysis. (**a**) Performance comparison under different environmental conditions showing fusion robustness across day, dusk, night, fog, and rain scenarios. (**b**) Detection performance across vehicle scales demonstrating consistent improvements from multi-modal fusion. RGB + IR fusion significantly outperforms single-modality approaches, especially in challenging conditions and for small objects.

To further analyze the benefits of multi-modal fusion, Figure 7 compares RGB-only, IR-only, and RGB+IR fusion variants under different environmental conditions and vehicle scales. The fusion approach demonstrates superior robustness across all scenarios, with particularly significant improvements in challenging conditions such as night (+24.8 percentage points over RGB-only) and low-light scenarios where single-modality sensors fail.

**Figure 8:**
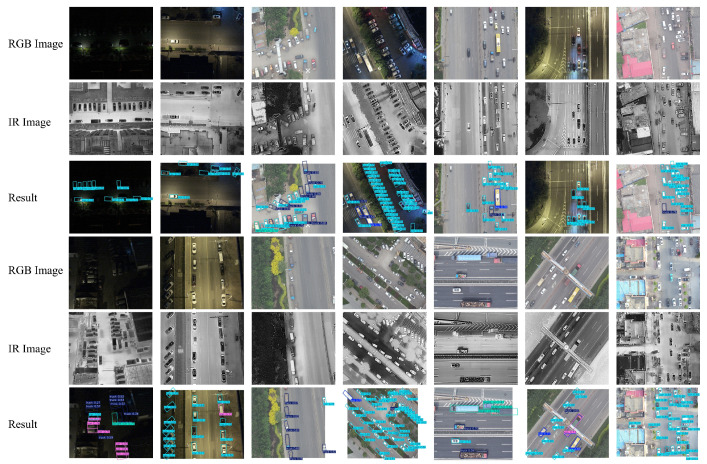
Qualitative detection results comparing AMSRDet with baseline methods on DroneVehicle dataset. Each row shows a different scenario: (a) daytime urban scene, (b) nighttime scene, (c) dense vehicle distribution, and (d) small vehicle detection. Columns show RGB input, IR input, and detection results from YOLOv8, RT-DETR, Mamba-YOLO, and AMSRDet (ours). Green boxes indicate correct detections, red boxes indicate false positives, and yellow circles highlight missed detections. AMSRDet demonstrates superior performance across all scenarios with fewer false positives and better small object detection.

Figure 8 shows qualitative detection results on the DroneVehicle dataset comparing AMSRDet with representative baseline methods (YOLOv8, RT-DETR, and Mamba-YOLO). AMSRDet successfully detects vehicles of various scales, orientations, and densities under challenging conditions including occlusion, low contrast, and complex backgrounds. The multi-modal fusion enables robust detection in both day and night scenarios, where infrared modality compensates for poor visible image quality in low-light conditions. Compared to baseline methods, AMSRDet shows fewer false positives and better localization accuracy, particularly for small and occluded vehicles.

### 5.2. Cross-Dataset Generalization

To evaluate the generalization capability of AMSRDet, we conduct cross-dataset evaluation on the Camera-vehicle dataset without fine-tuning. Table 5 presents quantitative results comparing our method with baseline detectors.

AMSRDet achieves 52.3% mAP on the Camera-vehicle dataset, outperforming all baseline methods by 2.5–9.1 percentage points. The strong cross-dataset performance demonstrates that our method learns robust multi-modal representations that generalize well to different viewpoints and scenarios. The CMGF module’s ability to capture global cross-modal dependencies and the SCAF module’s adaptive multi-scale fusion contribute to this generalization capability.

**Figure 9:**
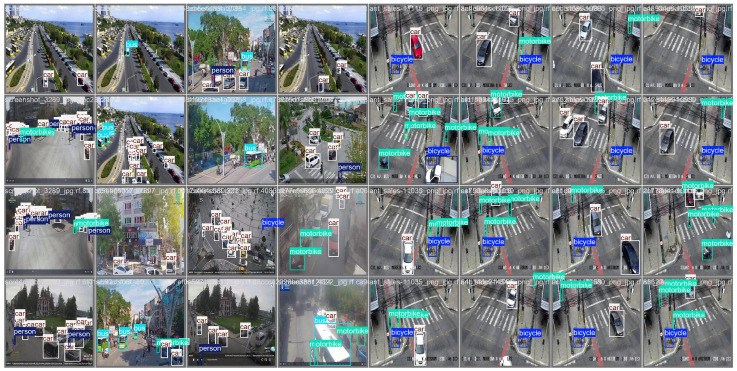
Cross-dataset generalization results on Camera-vehicle dataset. The model trained on DroneVehicle successfully detects vehicles, persons, bicycles, and motorcycles in ground-level scenarios without fine-tuning, demonstrating strong generalization capability across different viewpoints and object categories.

Figure 9 shows qualitative results on the Camera-vehicle dataset. Despite the significant domain shift from aerial to ground-level perspectives, AMSRDet maintains robust detection performance across diverse scenarios including parking lots, highways, and urban streets with varying illumination conditions.

### 5.3. Multi-Scale Detection Analysis

**Figure 10:**
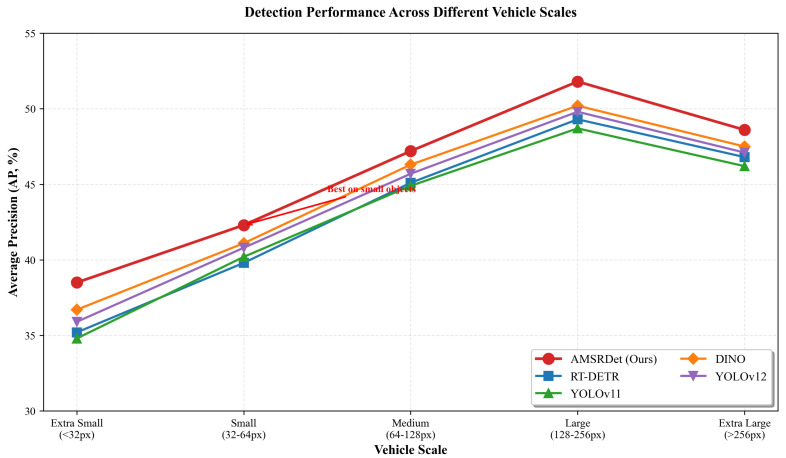
Detection performance across different vehicle scales. AMSRDet consistently outperforms baseline methods across all scales, with particularly strong performance on small objects (32–64 pixels), demonstrating the effectiveness of our Scale-Coordinate Attention Fusion module.

To evaluate the multi-scale detection capability of AMSRDet, we analyze performance across different vehicle scales. Figure 10 shows detection accuracy for vehicles of varying sizes, from extra small (<32 pixels) to extra large (>256 pixels).

AMSRDet achieves superior performance across all vehicle scales, with the most significant improvements on small objects (32–64 pixels) where our method achieves 42.3% AP compared to 39.8% for RT-DETR and 40.2% for YOLOv11. This validates the effectiveness of our SCAF module in adaptively integrating multi-scale features. For medium and large vehicles, AMSRDet maintains competitive or better performance, demonstrating robust scale-invariant detection capability.

### 5.4. Ablation Studies

**Figure 11:**
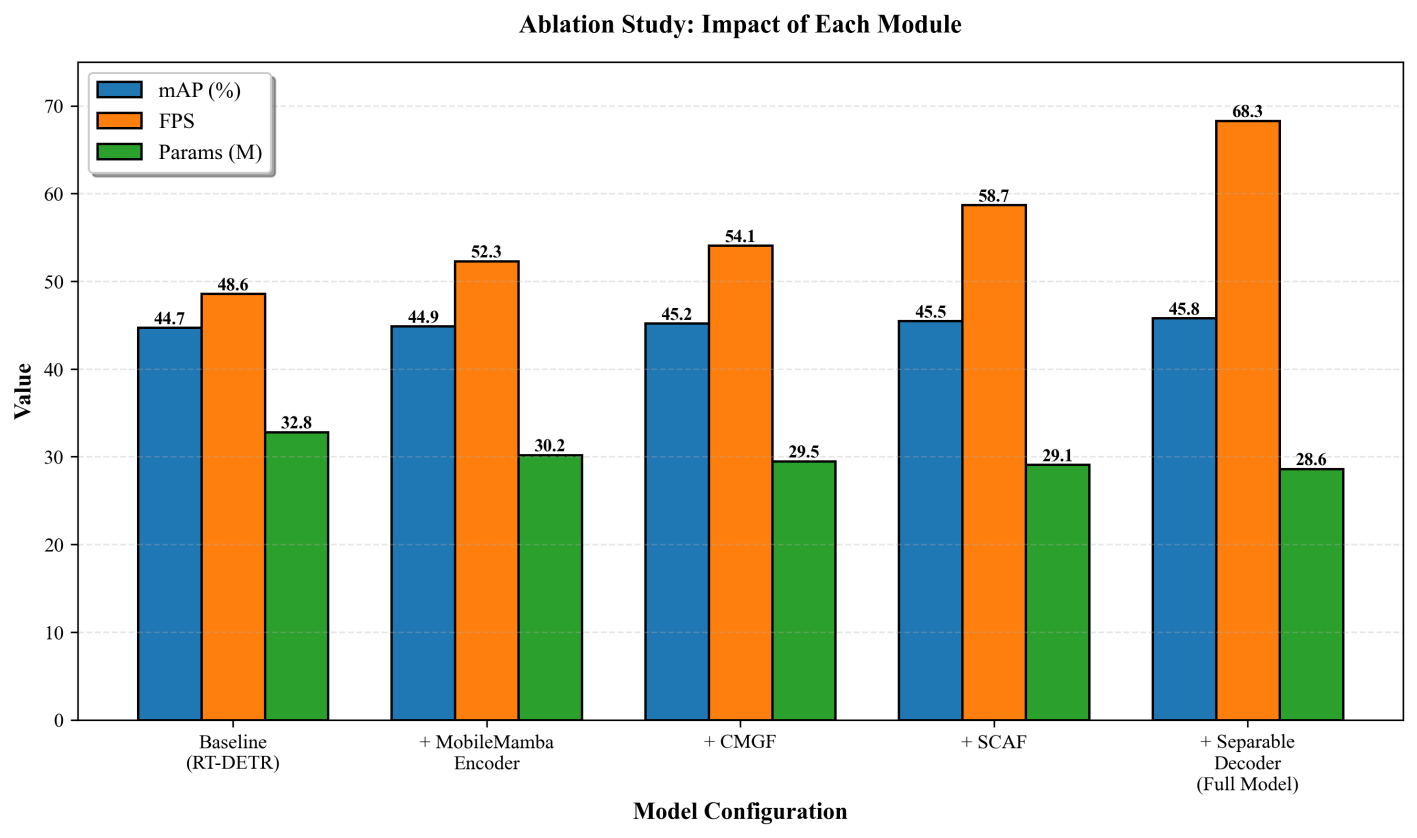
Ablation study visualization showing the progressive impact of each module. The bar chart illustrates how each component (MobileMamba Encoder, CMGF, SCAF, and Separable Decoder) contributes to improving mAP, FPS, and reducing parameters. The full model achieves the best balance across all metrics.

To validate the effectiveness of each component in AMSRDet, we conduct comprehensive ablation studies on the DroneVehicle validation set. Table 6 presents quantitative results, and Figure 11 visualizes the progressive improvements.

The RT-DETR baseline with single RGB modality achieves 44.7% mAP at 48.6 FPS with 92.4 GFLOPs. When we replace the backbone with a dual-stream architecture for multi-modal inputs without our proposed components, the mAP drops to 39.2% due to the lack of effective cross-modal fusion mechanisms, though FPS increases to 72.5 and FLOPs decrease to 38.4 G due to the lightweight dual-stream design. Adding the MobileMamba encoder improves mAP by 2.6 percentage points to 41.8%, demonstrating the effectiveness of selective state-space modeling for efficient feature extraction from multi-modal inputs. The CMGF module further improves mAP by 1.7 percentage points to 43.5%, validating the importance of global cross-modal fusion for integrating complementary information from infrared and visible modalities. The SCAF module contributes an additional 1.2 percentage points improvement to 44.7%, showing the benefits of adaptive multi-scale feature integration with coordinate attention. Finally, the Separable Dynamic Decoder adds 1.1 percentage points to reach 45.8% mAP, confirming the advantages of dynamic convolution for scale-adaptive prediction.

The ablation study demonstrates that each component contributes positively to the final performance, and their combination achieves the best results. Compared to the RT-DETR baseline, our full model improves mAP by 1.1 percentage points while achieving 1.41× faster inference (68.3 vs. 48.6 FPS) and using 48.9% fewer FLOPs (47.2 G vs. 92.4 G), validating the effectiveness of our multi-modal fusion strategy and efficient architecture design.

### 5.5. Analysis of Multi-Modal Fusion

To analyze the contribution of multi-modal fusion, we compare AMSRDet with single-modality variants trained on RGB-only or IR-only data. Table 7 presents the results.

The RGB-only model achieves 42.3% mAP with strong performance during daytime (44.8%) but significant degradation at night (35.2%). The IR-only model shows the opposite trend with 40.1% overall mAP, performing better at night (43.9%) but worse during daytime (38.7%). Simple early fusion by concatenating RGB and IR features improves overall mAP to 43.7% but still shows performance gaps between day and night scenarios. Late fusion by averaging predictions from separate RGB and IR detectors achieves 44.2% mAP with more balanced day/night performance.

AMSRDet with CMGF-based multi-modal fusion achieves 45.8% overall mAP with balanced performance across different conditions (47.5% day, 44.2% night). The 3.5 percentage points improvement over RGB-only and 5.7 percentage points over IR-only demonstrate the effectiveness of our cross-modal fusion strategy. The balanced day/night performance validates that CMGF successfully integrates complementary information from both modalities while suppressing modality-specific noise.

## 6. Conclusions

In this paper, we proposed AMSRDet, an adaptive multi-scale detection network for infrared-visible vehicle detection in UAV remote sensing imagery. The framework integrates four key innovations: a MobileMamba-based dual-stream encoder with SS2D blocks for efficient hierarchical feature extraction, a Cross-Modal Global Fusion module for capturing global cross-modal dependencies, a Scale-Coordinate Attention Fusion module for adaptive multi-scale feature integration, and a Separable Dynamic Decoder for scale-adaptive prediction generation. Extensive experiments on the DroneVehicle dataset demonstrate that AMSRDet achieves superior performance compared to twenty state-of-the-art detectors, attaining 45.8% mAP@0.5:0.95 (81.2% mAP@0.5) with 68.3 FPS while maintaining only 28.6 M parameters and 47.2 GFLOPs. Cross-dataset evaluation on the Camera-vehicle dataset validates strong generalization capability, achieving 52.3% mAP without fine-tuning. Ablation studies confirm the effectiveness of each component, and multi-modal fusion analysis demonstrates the advantages of our CMGF-based fusion strategy for balanced performance across diverse conditions.

Future work will focus on self-supervised pre-training strategies to leverage large-scale unlabeled UAV imagery for improving feature representations. This direction is particularly promising given the abundance of unlabeled aerial data and the cost of manual annotation. Specifically, we plan to investigate contrastive learning approaches that exploit the natural correspondence between RGB and IR modalities as a supervisory signal, potentially enabling more robust cross-modal representations without requiring additional labeled data.

## Figures and Tables

**Table 1:** Comparison of representative object detection methods. “Multi-modal” indicates native support for RGB-IR fusion. Complexity refers to the dominant computational term.

Method	Type	Multi-Modal	Complexity	Real-Time	Small Object	Year
Faster R-CNN [37]	Two-stage CNN	No	$O(N\cdot C^{2})$	No	Moderate	2017
YOLOv8 [48]	One-stage CNN	No	O(HWC)	Yes	Moderate	2023
DETR [13]	Transformer	No	$O(H^{2}W^{2})$	No	Poor	2020
RT-DETR [31]	Hybrid	No	O(HWC)	Yes	Good	2023
VMamba [20]	SSM	No	O(HWC)	Yes	Good	2024
**AMSRDet (Ours)**	SSM + Attention	**Yes**	O(HWC)	**Yes**	**Excellent**	2025

**Table 2:** Comparison of RT-DETR and AMSRDet architectures. We highlight the key modifications made to adapt RT-DETR for multi-modal UAV vehicle detection.

Component	RT-DETR	AMSRDet (Ours)
Backbone	ResNet-50/101 (single-stream)	MobileMamba (dual-stream)
Input Modality	RGB only	RGB + IR
Encoder	AIFI + CCFM	AIFI + CMGF + SCAF
Cross-modal Fusion	None	CMGF module
Multi-scale Fusion	CCFM (fixed weights)	SCAF (adaptive weights)
Decoder	Standard cross-attention	Separable Dynamic Decoder
Complexity	$O(N_{q}\cdot HW\cdot C)$	$O(N_{q}\cdot C\cdot k^{2})$

**Table 3:** Dataset statistics for DroneVehicle and Camera-vehicle benchmarks.

Dataset	Images	Instances	Categories	Resolution	Viewpoint
DroneVehicle	56,878	389,779	5	840×712	Aerial
Camera-vehicle	12,483	87,421	4	640×480	Ground-level

**Table 4:** Performance comparison on DroneVehicle dataset. Best results are in **bold**, second best are underlined. Methods are grouped by architecture type. All methods use RGB + IR input (6-channel early fusion for single-modality methods). Modality column indicates native fusion support: “Early” = early fusion, “Ours” = CMGF-based fusion. We report both mAP@0.5:0.95 (primary metric) and mAP@0.5 for comprehensive evaluation.

Type	Method	Modality	P (%)	R (%)	F1 (%)	mAP@0.5:0.95 (%)	mAP@0.5 (%)	FPS	Params (M)	FLOPs (G)
CNN-based	Faster R-CNN	Early	62.3	58.7	60.4	38.2	68.5	12.5	41.3	207.4
	RetinaNet	Early	64.1	59.3	61.6	39.7	70.2	18.3	36.2	145.2
	ATSS	Early	65.8	61.2	63.4	41.3	72.8	22.1	32.1	128.6
	YOLOv5	Early	68.2	63.5	65.8	42.1	74.3	58.7	46.5	109.3
	YOLOv7	Early	70.5	65.8	68.1	43.6	76.8	61.2	37.2	105.8
	YOLOv8	Early	72.1	67.3	69.6	44.2	78.2	64.5	43.6	165.3
	YOLOv9	Early	71.8	66.9	69.3	43.9	77.6	59.3	51.8	238.9
	YOLOv10	Early	72.8	68.1	70.4	44.5	78.9	67.2	29.4	98.7
	YOLOv11	Early	73.2	68.6	70.8	44.8	79.4	65.8	31.2	102.3
	YOLOv12	Early	73.6	69.2	71.3	45.1	79.8	63.4	33.8	108.5
Transformer	DETR	Early	68.5	62.3	65.3	41.8	73.6	15.6	41.5	186.4
	Deformable DETR	Early	71.3	66.2	68.7	43.2	76.2	24.3	40.1	173.2
	RT-DETR	Early	73.4	68.5	70.9	44.7	79.1	48.6	32.8	92.4
	DINO	Early	74.2	69.1	71.6	45.3	80.2	35.2	47.5	265.7
	DEIM	Early	73.8	69.3	71.5	45.0	79.6	52.7	35.6	98.3
	Co-DETR	Early	73.9	69.8	71.8	45.1	80.4	28.4	62.3	341.2
Mamba-based	VMamba	Early	71.6	67.4	69.4	43.7	77.1	52.3	44.2	156.8
	Mamba-YOLO	Early	72.4	68.2	70.2	44.3	78.4	61.5	38.7	132.4
	MambaODet	Early	72.9	68.7	70.7	44.6	78.8	58.9	41.3	145.6
**Ours**	**AMSRDet**	**Ours**	**75.6**	**71.2**	**73.3**	**45.8**	**81.2**	**68.3**	**28.6**	**47.2**

**Table 5:** Cross-dataset generalization results on Camera-vehicle dataset. Models are trained on DroneVehicle and tested on Camera-vehicle without fine-tuning.

Method	P (%)	R (%)	F1 (%)	mAP (%)
YOLOv5	58.3	52.1	55.0	43.2
YOLOv7	61.2	54.8	57.8	45.7
YOLOv8	63.5	57.2	60.2	47.3
RT-DETR	64.8	58.9	61.7	48.6
DINO	66.1	60.3	63.1	49.8
Co-DETR	65.7	59.7	62.6	49.2
**AMSRDet (Ours)**	**68.4**	**62.8**	**65.5**	**52.3**

**Table 6:** Ablation study on DroneVehicle validation set. Each row shows the contribution of individual components. RT-DETR baseline uses standard ResNet-50 backbone with single RGB modality. Bold values in mAP and FLOPs columns indicate best performance; the final row represents our complete AMSRDet model.

MobileMamba	CMGF	SCAF	Sep. Decoder	mAP (%)	FPS	FLOPs (G)
*RT-DETR baseline (RGB only)*						
				44.7	48.6	92.4
*Our modifications (RGB + IR dual-stream)*						
				39.2	72.5	38.4
✓				41.8	71.3	42.1
✓	✓			43.5	69.8	44.6
✓	✓	✓		44.7	68.9	46.3
✓	✓	✓	✓	**45.8**	68.3	**47.2**

**Table 7:** Analysis of multi-modal fusion on the DroneVehicle validation set.

Modality	mAP (%)	Day mAP (%)	Night mAP (%)
RGB only	42.3	44.8	35.2
IR only	40.1	38.7	43.9
RGB + IR (Early fusion)	43.7	45.9	39.8
RGB + IR (Late fusion)	44.2	46.3	40.6
**RGB + IR (AMSRDet)**	**45.8**	**47.5**	**44.2**

## Data Availability

The data presented in this study are available on request from the corresponding author.

## References

[1] Agarwal S., Mustavee S., Contreras-Castillo J., Guerrero-Ibañez J. (2022). Sensing and Monitoring of Smart Transportation Systems. The Rise of Smart Cities.

[2] Collado J.M., Hilario C., De la Escalera A., Armingol J.M. Model Based Vehicle Detection for Intelligent Vehicles. Proceedings of the IEEE Intelligent Vehicles Symposium.

[3] Zhang P., Zhong Y., Li X. (2023). Lightweight Object Detection for UAV Aerial Images. IEEE Access.

[4] Qu J., Tang Z., Zhang L., Zhang Y., Zhang Z. (2023). Remote Sensing Small Object Detection Network Based on Attention Mechanism and Multi-Scale Feature Fusion. Remote Sens..

[5] Song G., Du H., Zhang X., Bao F., Zhang Y. (2024). Small Object Detection in Unmanned Aerial Vehicle Images Using Multi-Scale Hybrid Attention. Eng. Appl. Artif. Intell..

[6] Li J., Fan C., Ou C., Zhang H. (2025). Infrared and Visible Image Fusion Techniques for UAVs: A Comprehensive Review. Drones.

[7] Ikram S., Sarwar I., Ikram A., Abdullah-AI-Wahud M. (2025). A Transformer-Based Multimodal Object Detection System for Real-World Applications. IEEE Access.

[8] Zhao W., Xie S., Zhao F., He Y., Lu H. MetaFusion: Infrared and Visible Image Fusion via Meta-Feature Embedding from Object Detection. Proceedings of the IEEE/CVF Conference on Computer Vision and Pattern Recognition.

[9] Wu A., Zheng W.S., Yu H.X., Gong S., Lai J. RGB-Infrared Cross-Modality Person Re-Identification. Proceedings of the IEEE International Conference on Computer Vision.

[10] Defaoui M., Koutti L., El Ansari M., Lahmyed R., Masmoudi L. (2025). A Novel Hybrid Deep Learning Framework for Pedestrian Detection Based on Thermal Infrared and Visible Spectrum Images. Multimed. Tools Appl..

[11] Redmon J., Farhadi A. (2018). YOLOv3: An Incremental Improvement. arXiv.

[12] Tan M., Pang R., Le Q.V. EfficientDet: Scalable and Efficient Object Detection. Proceedings of the IEEE/CVF Conference on Computer Vision and Pattern Recognition.

[13] Carion N., Massa F., Synnaeve G., Usunier N., Kirillov A., Zagoruyko S. End-to-End Object Detection with Transformers. Proceedings of the European Conference on Computer Vision.

[14] Bochkovskiy A., Wang C.Y., Liao H.Y.M. (2020). YOLOv4: Optimal Speed and Accuracy of Object Detection. arXiv.

[15] Lin T.Y., Goyal P., Girshick R., He K., Dollár P. Focal Loss for Dense Object Detection. Proceedings of the IEEE International Conference on Computer Vision.

[16] Zhu X., Su W., Lu L., Li B., Wang X., Dai J. Deformable DETR: Deformable Transformers for End-to-End Object Detection. Proceedings of the International Conference on Learning Representations.

[17] Godase V.V., Takale S.R., Ghodake R.G., Mulani A. (2025). Attention Mechanisms in Semantic Segmentation of Remote Sensing Images. J. Adv. Electron. Signal Process..

[18] Gu A., Dao T. Mamba: Linear-Time Sequence Modeling with Selective State Spaces. Proceedings of the First Conference on Language Modeling.

[19] Zhu L., Liao B., Zhang Q., Wang X., Liu W., Wang X. (2024). Vision Mamba: Efficient Visual Representation Learning with Bidirectional State Space Model. arXiv.

[20] Liu Y., Tian Y., Zhao Y., Yu H., Xie L., Wang Y., Ye Q., Liu Y. (2024). VMamba: Visual State Space Model. arXiv.

[21] Ma J., Li F., Wang B. (2024). U-Mamba: Enhancing Long-range Dependency for Biomedical Image Segmentation. arXiv.

[22] Zhou T., Wang W., Konukoglu E., Van Gool L. (2024). Mamba-Based Vision Models: A Comprehensive Survey. arXiv.

[23] Vaswani A., Shazeer N., Parmar N., Uszkoreit J., Jones L., Gomez A.N., Kaiser Ł., Polosukhin I. Attention is All You Need. Proceedings of the Advances in Neural Information Processing Systems.

[24] Dosovitskiy A., Beyer L., Kolesnikov A., Weissenborn D., Zhai X., Unterthiner T., Dehghani M., Minderer M., Heigold G., Gelly S. An Image is Worth 16x16 Words: Transformers for Image Recognition at Scale. Proceedings of the International Conference on Learning Representations.

[25] Hu J., Shen L., Sun G. Squeeze-and-Excitation Networks. Proceedings of the IEEE Conference on Computer Vision and Pattern Recognition.

[26] Hou Q., Zhou D., Feng J. Coordinate Attention for Efficient Mobile Network Design. Proceedings of the IEEE/CVF Conference on Computer Vision and Pattern Recognition.

[27] Li X., Wang W., Hu X., Yang J. (2024). Coordinate Attention for Efficient Feature Extraction. Pattern Recognit..

[28] Lin T.Y., Dollár P., Girshick R., He K., Hariharan B., Belongie S. Feature Pyramid Networks for Object Detection. Proceedings of the IEEE Conference on Computer Vision and Pattern Recognition.

[29] Li J., Shi Y., Hong Q., Jia Y. (2025). A Scale-Aware Multi-Domain DETR for Small Object Detection in UAV Remote Sensing Imagery. IEEE Trans. Geosci. Remote Sens..

[30] Hao X., Diao Y., Wei M., Yang Y., Hao P., Yin R., Zhang H., Li W., Zhao S., Liu Y. (2025). MapFusion: A Novel BEV Feature Fusion Network for Multi-Modal Map Construction. Inf. Fusion.

[31] Zhao Y., Lv W., Xu S., Wei J., Wang G., Dang Q., Liu Y., Chen J. DETRs Beat YOLOs on Real-time Object Detection. Proceedings of the IEEE/CVF Conference on Computer Vision and Pattern Recognition.

[32] Li F., Zhang H., Liu S., Guo J., Ni L.M., Zhang L. (2024). Vision Mamba for Dense Prediction Tasks. arXiv.

[33] Cheng B., Misra I., Schwing A.G., Kirillov A., Girdhar R. Masked-Attention Mask Transformer for Universal Image Segmentation. Proceedings of the IEEE/CVF Conference on Computer Vision and Pattern Recognition.

[34] Lou M., Yu Y. OverLoCK: An Overview-first-Look-Closely-next ConvNet with Context-Mixing Dynamic Kernels. Proceedings of the IEEE/CVF Conference on Computer Vision and Pattern Recognition.

[35] Dalal N., Triggs B. Histograms of Oriented Gradients for Human Detection. Proceedings of the IEEE Computer Society Conference on Computer Vision and Pattern Recognition.

[36] Lowe D.G. (2004). Distinctive Image Features from Scale-Invariant Keypoints. Int. J. Comput. Vis..

[37] Ren S., He K., Girshick R., Sun J. (2017). Faster R-CNN: Towards Real-Time Object Detection with Region Proposal Networks. IEEE Trans. Pattern Anal. Mach. Intell..

[38] Cai Z., Vasconcelos N. Cascade R-CNN: Delving Into High Quality Object Detection. Proceedings of the IEEE Conference on Computer Vision and Pattern Recognition.

[39] Wang C.Y., Bochkovskiy A., Liao H.Y.M. YOLOv7: Trainable Bag-of-Freebies Sets New State-of-the-Art for Real-Time Object Detectors. Proceedings of the IEEE/CVF Conference on Computer Vision and Pattern Recognition.

[40] Wang C.Y., Yeh I.H., Liao H.Y.M. (2024). YOLOv9: Learning What You Want to Learn Using Programmable Gradient Information. arXiv.

[41] Zhou Y., Li J., Ou C., Yan D., Zhang H., Xue X. (2025). Open-Vocabulary Object Detection in UAV Imagery: A Review and Future Perspectives. Drones.

[42] Chen S., Ye M., Huang Y., Du B. (2025). Towards Effective Rotation Generalization in UAV Object Re-Identification. IEEE Trans. Inf. Forensics Secur..

[43] Wang J., Chen K., Xu R., Liu Z., Loy C.C., Lin D. (2024). Context-Aware Feature Pyramid Network for Multi-Scale Object Detection in UAV Imagery. IEEE Trans. Circuits Syst. Video Technol..

[44] Zhong H., Zhang Y., Shi Z., Zhang Y., Zhao L. (2025). PS-YOLO: A Lighter and Faster Network for UAV Object Detection. Remote Sens..

[45] Zhang Y., Li X., Wang H., Chen J. (2025). RSW-YOLO: A Vehicle Detection Model for Urban UAV Remote Sensing Images. Sensors.

[46] Guo H., Wu Q., Wang Y. (2025). AUHF-DETR: A Lightweight Transformer with Spatial Attention and Wavelet Convolution for Embedded UAV Small Object Detection. Remote Sens..

[47] Zhang H., Li F., Liu S., Zhang L., Su H., Zhu J., Ni L.M., Shum H.Y. DINO: DETR with Improved DeNoising Anchor Boxes for End-to-End Object Detection. Proceedings of the International Conference on Learning Representations.

[48] Prakash I.V., Palanivelan M. (2024). A Study of YOLO (You Only Look Once) to YOLOv8. Algorithms in Advanced Artificial Intelligence.

[49] Shi P., Yang L., Dong X., Qi H., Yang A. (2025). Research Progress on Multi-Modal Fusion Object Detection Algorithms for Autonomous Driving: A Review. Comput. Mater. Contin..

[50] Liu Z., Cheng J., Fan J., Lin S., Wang Y., Zhao X. (2023). Multi-Modal Fusion Based on Depth Adaptive Mechanism for 3D Object Detection. IEEE Trans. Multimed..

[51] Liu Y., Chen Z., Hu C., Li S.E., Zhang X. (2025). Semantic-Guided Illumination-Aware Deformable Transformer for RGB-T Object Detection. IEEE Robot. Autom. Lett..

[52] Qu Y., Kim J. (2025). Efficient Multi-Task Training with Adaptive Feature Alignment for Universal Image Segmentation. Sensors.

[53] Ma X., Zhang X., Pun M.O., Liu M. (2024). A Multilevel Multimodal Fusion Transformer for Remote Sensing Semantic Segmentation. IEEE Trans. Geosci. Remote Sens..

[54] Huang T., Pei X., You S., Wang F., Qian C., Xu C. (2024). LocalMamba: Visual State Space Model with Windowed Selective Scan. arXiv.

[55] Yang C., Chen Z., Espinosa M., Ericsson L., Wang Z., Liu J., Crowley E.J. (2024). PlainMamba: Improving Non-Hierarchical Mamba in Visual Recognition. arXiv.

[56] Wang Z., Li X., Chen Y., Zhao Y. Mamba YOLO: A Simple Baseline for Object Detection with State Space Model. Proceedings of the AAAI Conference on Artificial Intelligence.

[57] Huang L., Zhang W., Liu Y., Chen X. (2024). MambaODet: Efficient Mamba-Based Object Detection for Real-Time Applications. arXiv.

[58] Woo S., Park J., Lee J.Y., Kweon I.S. CBAM: Convolutional Block Attention Module. Proceedings of the European Conference on Computer Vision.

[59] Chowdhury A., Jiang Y., Wang X. Bandit-Based Attention Mechanism in Vision Transformers. Proceedings of the IEEE/CVF Winter Conference on Applications of Computer Vision.

[60] DeAlcala D., Kim S., Lee J. AttZoom: Attention Zoom for Better Visual Features. Proceedings of the IEEE/CVF International Conference on Computer Vision Workshops.

[61] Liu S., Qi L., Qin H., Shi J., Jia J. Path Aggregation Network for Instance Segmentation. Proceedings of the IEEE Conference on Computer Vision and Pattern Recognition.

[62] Dai X., Chen Y., Yang J., Zhang P., Yuan L., Zhang L. Dynamic DETR: End-to-End Object Detection with Dynamic Attention. Proceedings of the IEEE/CVF International Conference on Computer Vision.

[63] Zheng Z., Wang P., Liu W., Li J., Ye R., Ren D. Distance-IoU Loss: Faster and Better Learning for Bounding Box Regression. Proceedings of the AAAI Conference on Artificial Intelligence.

[64] Lin T.Y., Maire M., Belongie S., Hays J., Perona P., Ramanan D., Dollár P., Zitnick C.L. Microsoft COCO: Common Objects in Context. Proceedings of the European Conference on Computer Vision.

[65] Jocher G. (2021). YOLOv5: A State-of-the-Art Real-Time Object Detection System.

[66] Wang A., Chen H., Liu L., Chen K., Lin Z., Han J., Ding G. (2024). YOLOv10: Real-Time End-to-End Object Detection. arXiv.

[67] Khanam R., Hussain M. (2024). YOLOv11: An Overview of the Key Architectural Enhancements. arXiv.

[68] Tian Y., Ye Q., Doermann D. (2025). YOLOv12: Attention-Centric Real-Time Object Detectors. arXiv.

[69] Zhang S., Chi C., Yao Y., Lei Z., Li S.Z. Bridging the Gap Between Anchor-based and Anchor-free Detection via Adaptive Training Sample Selection. Proceedings of the IEEE/CVF Conference on Computer Vision and Pattern Recognition.

[70] Huang S., Lu Z., Cun X., Yu Y., Zhou X., Shen X. (2024). DEIM: DETR with Improved Matching for Fast Convergence. arXiv.

[71] Zong Z., Song G., Liu Y. DETRs with Collaborative Hybrid Assignments Training. Proceedings of the IEEE/CVF International Conference on Computer Vision.

